# In this month’s *Bulletin*

**DOI:** 10.2471/BLT.14.000914

**Published:** 2014-09-01

In editorials*,* Ruediger Krech & Marie-Paule Kieny (622) discuss the ethical implications of testing treatments for Ebola virus disease. Oleg Chestnov et al. (623) explain why noncommunicable disease control is integral to development goals.

Jane Parry (626–627) reports on China’s moves to produce vaccines for the global market. Bruno Vellas (628–629) talks to Fiona Fleck about identifying frail elderly patients in order to improve their quality of life.

## Austria, Estonia, Finland, Greece, Ireland, Portugal, Slovakia & Spain

## Responding to instability

Christine Leopold et al. (630–640) examine the effects of economic recession on pharmaceutical pricing policies.

## Albania, Latvia, Lithuania, Montenegro, Romania, the Russian Federation, The former Yugoslav Republic of Macedonia & Turkey

## Detecting late effects

Mark A Bellis et al. (641–655) examine correlations between harmful behaviour and adverse childhood experiences.

## China

## Improving the signal to noise ratio

Zhongjie Li et al. (656–663) evaluate an early warning system for hand, foot and mouth disease.

## Bangladesh, Ethiopia, Ghana, India, Pakistan, Uganda, the United Republic of Tanzania

## A persistent killer

Ahmed Ehsanur Rahman et al. (664–671) examine a decade of child deaths from diarrhoea.

## Mozambique

## Bringing care to patients

Troy D Moon et al. (680–684) describe the use of a mobile clinic for antiretroviral therapy.

## Brazil

## Improving vector control

Rafael Maciel-de-Freitas & Denise Valle (685–689) explain lessons learnt in trying to control *Aedes aegypti.*

## Malawi

## Overcoming the lack of a neighbourhood map

Veronica Escamilla et al. (690–694) use satellite imagery to identify households.

## Global

## Changing definitions

Susan D Cochran et al. (672–679) discuss declassification of disease categories related to sexual orientation.

## Ensuring adverse events are reported

Christine G Maure et al. (695–696) introduce the Global Vaccine Safety Initiative.

